# Comparison of the microbial, physicochemical, and sensorial properties of raw and pasteurized Lighvan cheeses during ripening time

**DOI:** 10.1002/fsn3.2511

**Published:** 2021-08-07

**Authors:** Parisa Rashtchi, Ali Bazmi, Nooshin Noshirvani, Mir Hassan Moosavy

**Affiliations:** ^1^ Department of Food Science and Technology Faculty of Agriculture University of Tabriz Tabriz Iran; ^2^ R & D Project Manager Cintech 3224 rue Sicotte St‐Hyacinthe Quebec Canada; ^3^ Department of Food Science and Technology Tuyserkan Faculty of Engineering & Natural Resources Bu‐Ali Sina University Hamedan Iran; ^4^ Department of Food Hygiene & Quality Control Faculty of Veterinary Medicine University of Tabriz Tabriz Iran

**Keywords:** Lighvan cheese, microbiological characteristics, pasteurized milk, physicochemical characteristics, raw milk

## Abstract

Traditional cheeses which are normally produced from raw milk are very popular due to their intense and unique taste and aroma. However, high microbial contamination of raw milk due to manual milking and secondary contamination may lead to many diseases in humans in Iran. Lighvan is a traditional starter‐free locally made Iranian cheese that is made from raw ewe's milk. Since the use of raw milk in the preparation of cheese produces serious health problems, due to the limited ripening period of this type of cheese, this study aimed to evaluate the feasibility of preparing Lighvan cheese from pasteurized milk. For this purpose, different characteristics of cheese prepared with pasteurized milk were compared with raw milk cheese. The results showed a reduction in the microbial population over the ripening time in both types of cheeses. However, coliforms and *Escherichia coli* were seen in raw milk cheeses until the last day of ripening. Regarding chemical analyses, the water‐soluble nitrogen fraction and lipolysis products increased during ripening. Moreover, the raw milk cheeses indicated a higher lipolysis index than the pasteurized ones. According to the obtained results from the sensory evaluation, the raw milk cheese indicated higher acceptability compared with the pasteurized milk cheese. However, since the presence of *E*. *coli* makes the cheese inedible, it seems that the pasteurization of milk is mandatory for the production of this type of cheese.

## Graphical abstract

- Liqhvan is a traditional Iranian cheese made from raw milk and is highly contaminated, especially coliforms and *E*. *coli*.
- Pasteurized milk cheese was prepared and examined whether this cheese could be a suitable substitute for raw cheese.
- Comparison of the physicochemical, microbiological, and sensory properties of raw and pasteurized cheeses was performed.
- Coliforms and *E.coli* were detected in raw cheese until the last day of ripening.
- Pasteurization of cheese milk is mandatory to ensure the safety of Lighvan cheese.

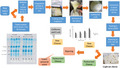

**Practical Implications**

Lighvan is the most popular cheese among all traditional cheeses in Iran, which is produced from raw milk. In Iran, a great extent of milk is produced in small traditional farms, and due to hand‐milking in most cases, the possibility of cross‐contamination is high in raw milk handling. Since maintaining the safety of raw cheese is associated with risks, regarding the importance of this issue, there is a need for extensive research in this area where limited studies are reported for this type of food product. In this study, pasteurized milk cheese was prepared and examined whether this cheese could be a suitable substitute for raw cheese. The comparison of the physicochemical, microbiological, and sensory properties of raw and pasteurized cheeses was performed. Coliforms and *E*. *coli* were detected in raw cheese until the last day of ripening. Accordingly, pasteurization of cheese milk is mandatory to ensure the safety of Lighvan cheese.

## 1 INTRODUCTION

Cheese is a highly appreciated dairy product of most people all over the world. Traditional cheeses which are normally produced from raw milk are very popular due to their intense and unique taste and aroma, as well as high nutritional value. However, several diseases related to the consumption of raw milk cheese have reported in the United States and other parts of the world. For example, many cases of death have been reported in raw milk cheeses contaminated with *E*. *coli* in the United States, Canada, and Europe. Therefore, raw milk cheese is one of the risky products (Yoon et al., 2016). Various foodborne bacteria including *Brucella melitensis*, *Campylobacter* spp., *Coxiella burnetii*, *E*. *coli* O157:H7, *Listeria monocytogenes, Mycobacterium* bovis, *Salmonella*, *Staphylococcus aureus*, and *Streptococcus* spp. are associated with raw milk cheese (Yoon et al., 2016). The growth of pathogens is a hazardous point in the safety and quality control of raw milk cheese, which mainly depends on the kind of cheese and its manufacturing technology.

Lighvan is a salty, starter‐free, and semi‐hard cheese made traditionally from a mixture of ewe's and goat's milk in Lighvan, a region of East Azerbaijan province in Iran. Due to the special taste and flavor, Lighvan is the most popular cheese among all traditional cheeses in Iran in which, and the amount of cheese production in the Lighvan region is 3,150 tons annually (Moosavy et al., 2014). The most important distinction between Lighvan cheese and other similar products is the use of raw milk without adding a starter culture in its formulation. Since in Iran, a great extent of milk is produced in small traditional farms, and due to hand‐milking in most cases, the possibility of cross‐contamination is high in raw milk handling (Hanifian & Khani, 2012). Mirzaei et al. (2008) collected 178 Lighvan cheese samples from Tabriz region and observed microbial contamination in some samples above the allowed limit of national standard for Iranian industrial white ripened cheese. Also, *E*. *coli*, fecal coliforms, and positive coagulase *S. aureus* were detected from 58.4, 62.9, and 18% of the cheeses, respectively. They concluded that the Lighvan cheese has a pleasant aroma and taste, but its production does not have satisfactory health conditions. In another research by Moosavy et al. (2014), the presence of *L*. *monocytogenes* was reported in 50% of raw milk samples utilized for the preparation of Lighvan cheese with 40 CFU/ml concentration. Besides that, according to previous studies (Fallah et al., 2011; Mohajeri et al., 2013; Shahbazi et al., 2017; Tavakoli et al., 2012), cheese is a potent source of Aflatoxin among dairy products due to the affinity of casein fraction to Aflatoxin, where the concentration of Aflatoxin in cheese is 3–5 time higher than the milk with more than 60% frequency of contamination.

At small dairies, the cheese is usually made from raw milk or the pasteurization process is applied by batch processing, which is not enough to kill all undesired microorganisms. In addition, in some countries, there are strict rules for the production or import of raw milk cheeses (Ryser, 2012). Nowadays, due to common diseases between human and animals, milk pasteurization is mandatory following international standards such as the Codex Alimentarius Commission (CAC) (1982). According to US regulations, cheese producers are required to choose one of the following to ensure the safety of the product from contamination: (1) pasteurization of milk or, (2) storage of milk at low temperature for at least 60 days (Pellegrino & Donnelly, 2004; Boor et al., 2017).

Considering the growing interest in the consumption of Lighvan cheese among consumers, to produce a product by international standards that can be exported to other countries and increase economic productivity, finding appropriate solutions is necessary. Despite the popularity of Lighvan cheese in the community and increasing its consumption among people, there are still few studies on the chemical (Aminifar et al., 2010; Lavasani et al., 2012) and microbiological (Hanifian & Khani, 2012) properties of this type of cheese. Mirzaei (2011) measured the number of microorganisms in Lighvan cheese during the ripening stage and claimed a large number of different microorganisms involved over ripening, which makes it more necessary to pay attention to hygiene matters in cheese making. To maintain public health, this study aimed to prepare Lighvan cheese from pasteurized milk and compare the physicochemical and sensorial properties of cheese made with raw milk and the feasibility of replacing cheese made with ordinary cheese.

## 2 MATERIALS AND METHODS

### 2.1 Cheese manufacture

**FIGURE 1:**
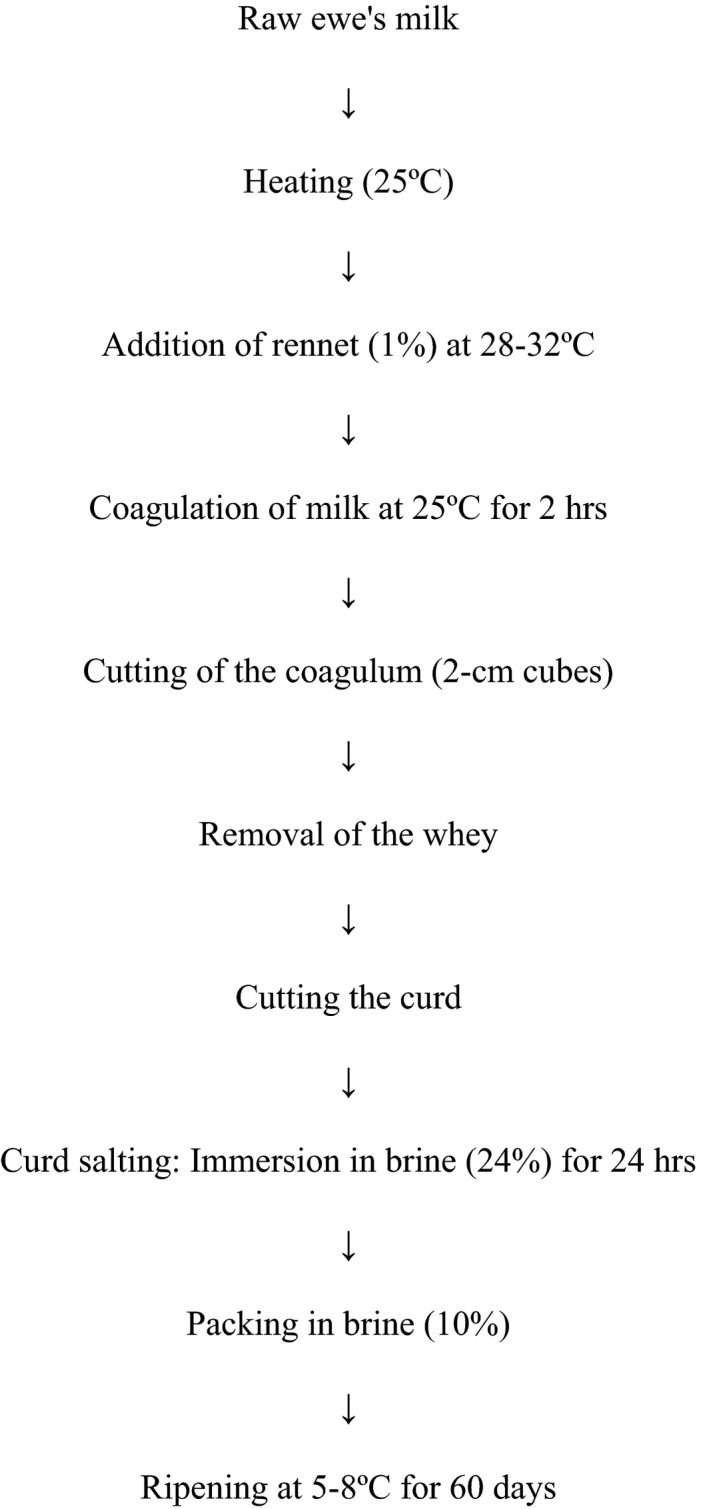
Diagram of manufacturing stages of Lighvan cheese

The raw ewe's milk was supplied from Lighvan in the province of Azerbaijan, Iran. The protocol for cheese manufacture is illustrated in Figure 1. The rennet was purchased from Metoo Co. (Japan) and added to the heated milk at 1% at 28‐32ºC, and then, the samples were kept for coagulation of milk at 25ºC for 2 hr. The coagulum was cut into 2‐cm cubes and kept until the removal of whey. Afterwards, the curd was cut again and immersed in brine (24%) for 24 hr. The obtained curd was packed in brine (10% W/V) and stored for 60 days at 5‐8ºC. For the pasteurized milk cheeses, the pasteurization was applied by low‐temperature long time (LTLT) at 63ºC for 30 min on raw milk, followed by cooling the milk to 25ºC.

### 2.2 Cheese analyses

The analyses were performed on the cheeses at 2, 14, 35, and 60 days of ripening. Both raw and pasteurized cheeses were analyzed for their fat (AOAC, 2000), the moisture content (Oven‐drying method at 102ºC (IDF Standard, 1982), and pH using a pH‐meter (model 632 Metrohm, Switzerland) by placing the pH‐meter electrode directly into grated cheese.

### 2.3 Proteolysis assessment

#### 2.3.1 The pH 4.6 soluble nitrogen

The method of Kuchroo and Fox (1982) was applied for the evaluation of pH 4.6 soluble of nitrogen extracts.

#### 2.3.2 Soluble nitrogen in trichloroacetic acid (TCA)

The Macro‐Kjeldahl method (IDF standard 20B, 1993) was applied for the measurement of the nitrogen content of cheeses.

#### 2.3.3 Electrophoresis

The urea‐polyacrylamide gel electrophoresis (PAGE) was applied to analyze the cheese samples, and the water‐soluble extracts were assessed based on the Shalabi and Fox (1987).

### 2.4 Lipolysis assessment

The Nunez et al. (1986) was used to determine free fatty acids.

### 2.5 Microbiological analyses

Before microbiological tests, 10 g of each cheese sample was homogenized in 90 mL of sterile sodium citrate solution with pulsifier, followed by setting up the decimal dilutions in sterile peptone (0.1%) water. The microbiological analysis of milk and cheese samples was performed by counting the following microorganisms:

- Total bacteria on plate count agar (PCA) medium (Merck, Germany) incubated for 72 hr at 30ºC;
- Thermoduric bacteria after heat treatment of milk at 63ºC for 30 min on (PCA) medium (Merck, Germany) incubated for 72 hr at 30ºC;
- Coliforms on violet red bile agar (Merck, Germany) incubated for 24 hr at 37ºC;
- *Enterococci* on kanamycin aesculin azide agar medium (Merck, Germany) incubated for 48 hr at 37ºC;
- *Micrococcacea* on mannitol salt agar (Merck, Germany) medium incubated for 72 hr at 30ºC;
- Lactic acid bacteria on MRS agar medium (Merck, Germany) incubated for 72 hr at 30ºC;
- *Lactobacillus* on rogosa agar (Merck, Germany) incubated an aerobically at 37ºC for 48 hr;
- Yeast on DRCM agar incubated for 72 hr at 25ºC;
- *E*. *coli* was identified according to the methods of the American Public Health Association (1972);
- *L*. *monocytogenes* on PALCAM Listeria agar selective agar base (Merck, Germany) with the selective supplement for PALCAM (Merck, Germany) incubated at 37ºC for 48 hr (Arques et al., 2005);
- *Salmonella* identified on the basis of the methods of Poelma et al. (1977).

### 2.6 Sensory assessment

The 10‐point hedonic test was used for the sensory evaluation of cheese samples. 15 trained panelists evaluated the flavor and texture of two types of raw and pasteurized cheese on the last day of storage with the scale of 1 = poor to 100 = excellent.

### 2.7 Statistical analysis

The obtained data were an average of 3 replicates per sample. The analysis of variance using SAS (STAT 9.2) software was performed for statistical analysis of the data. Significant differences at *p* < .05 were investigated.

## 3 RESULTS AND DISCUSSION

### 3.1 Microbiological characteristics

The results of microbiological tests on the dominant microbes of both cheeses during the storage period are shown in Table 1. Cheese samples prepared from raw milk showed high contamination (9.19 log CFU/gr) on the first day, which indicates severe microbial contamination of sheep's milk in the Lighvan region. However, during the ripening period, the total bacteria counts decreased significantly (*p* < .05) and reached to 7.26 log CFU/gr after 60 days of ripening time. The reduction in the microbial counts over the ripening time is attributed to the development of inhibitory conditions such as low pH, deficiency of oxygen, the presence of salt, and the relatively low temperature of storage. Also, depletion of lactose by lactic acid bacteria and the activity of the secondary microflora can be effective in microbial load (Mirzaei, 2011).

**TABLE 1:** Counts of the main selected microbial groups, expressed as Log CFU/g over the ripening of Lighvan cheese

Microflora	Cheese type	Ripening time (day)			
		2	14	35	60
Total bacteria	Raw	9.19 ± 0.97^a^	8.82 ± 0.07^b^	7.81 ± 0.07^c^	7.26 ± 0.07^d^
	Pasteurized	6.60 ± 0.07^a^	5.86 ± 0.07^b^	5.21 ± 0.07^c^	4.81 ± 0.07^d^
Coliforms	Raw	5.95 ± 0.16^a^	4.73 ± 0.016^b^	3.40 ± 0.15^c^	2.72 ± 0.15^d^
	Pasteurized	‐	‐	‐	‐
*Enterococci*	Raw	7.05 ± 0.17^a^	6.83 ± 0.14^b^	6.40 ± 0.17^c^	5.87 ± 0.14^d^
	Pasteurized	4.80 ± 0.14^a^	4.51 ± 0.17^b^	4.03 ± 0.14^d^	4.1 ± 0.14 cd
Thermoduric bacteria	Raw	2.73 ± 0.49^b^	4.56 ± 0.49^a^	2.98 ± 0.49^d^	3.26 ± 0.49^c^
	Pasteurized	1.87 ± 0.6^d^	2.03 ± 0.49^c^	3.22 ± 0.49^a^	2.32 ± 0.6^b^
Micrococcaceae	Raw	5.72 ± 0.1^b^	6.63 ± 0.12^a^	6.73 ± 0.1^a^	6.68 ± 0.12^a^
	Pasteurized	5.58 ± 0.12^a^	5.39 ± 0.12^a^	5.56 ± 0.14^a^	5.55 ± 0.1^a^
*Lactobacilli*	Raw	6.78 ± 0.04^a^	6.64 ± 0.04^ab^	5.49 ± 0.06^d^	5.78 ± 0.04^c^
	Pasteurized	‐	‐	‐	‐
Yeast	Raw	3.78 ± 0.05^a^	3.65 ± 0.06^ab^	3.75 ± 0.06^ab^	3.08 ± 0.06^c^
	Pasteurized	‐	‐	‐	‐
Lactic acid bacteria	Raw	8.17 ± 0.09^a^	7.86 ± 0.09^b^	6.74 ± 0.09^c^	6.67 ± 0.09^c^
	Pasteurized	3.66 ± 0.09^a^	3.76 ± 0.1^a^	3.77 ± 0.1^a^	3.72 ± 0.09^a^
*E*. *coli*	Raw	+	+	+	+
	Pasteurized	‐	‐	‐	‐
*Listera*	Raw	1.47^a^	1^b^	‐	‐
	Pasteurized	‐	‐	‐	‐
*Salmonella*	Raw	‐	‐	‐	‐
	Pasteurized	‐	‐	‐	‐

^(a,b)^ Means in the same raw, the same superscript does not differ significantly at *p* < .05.

The numbers of coliforms and *Enterococci* reduced significantly (*p* < .05) over ripening time in cheese samples made from raw milk from 5.95 log CFU/gr to 2.72 log CFU/gr and 7.05 log CFU/gr to 5.85 log CFU/gr, respectively. The raw milk cheese indicated the high counts of coliforms known as hygiene indicator microorganisms, attributed to the milk contamination during milking, shipping, and manufacturing. The overall growth of coliforms is related to the early days of storage and under appropriate environmental conditions especially, before acidification. The amount of *Micrococcus* reached to 6.63 log CFU/gr after 14 days, and afterward, did not change significantly *(p* < .05) in raw milk cheeses. Due to lipolytic and proteolytic activities, *Micrococcus* has an important role in cheese ripening. Lactic acid bacteria count decreased significantly until 14 days (*p* < .05), but subsequently, its population was stable. Results confirm that *Lactobacillus* counts decreased over the ripening period, but no significant difference was seen between days 2 and 14 (*p* > .05). The number of yeasts was approximately stable over the ripening time, and a considerable difference was observed only between days 35 and 60. The occurrence of yeasts in cheese with a stable population until the last day of ripening is attributed to the tolerance to severe environmental conditions such as low pH, decreased water activity, and high salt concentration, and thus, they appear to affect organoleptic properties of cheese due to lipolytic and proteolytic activities. However, Mirzaei (2011) reported a decrease in yeast population during ripening time in goat and Lighvan cheeses. *L.monocytogenes, Salmonella, E*. *coli, and Campylobacter jejuni* are the most important pathogens in milk. In this work, the effect of the type of treatment and the duration of cheese ripening on the growth of *L*. *monocytogenes, Salmonella, and E*. *coli* was investigated. None of these three bacteria were found in pasteurized cheeses. *Salmonella* was never seen in any of cheeses, and not in cheese milk either. *L*. *monocytogenes* was present in raw cheese at a very lower number than the other major microbial groups (1 log CFU/gr at 14th day of storage time) and disappeared after 35 days. Inactivation of *L*. *monocytogenes* in cheeses made from raw milk after 14 days is related to the antagonistic function of LAB as well as raising the NaCl concentration over the storage. However, *E*. *coli* was observed in raw milk cheeses even after 60 days of storage in the salty brine. Similarly, Vaziri and Naghshbandi (2011) in a study evaluated the contamination of local Lighvan cheese to coliforms and *E*. *coli* in the Maragheh city in East Azerbaijan province of Iran. Based on their findings, the incidence of coliforms and *E*. *coli* in samples were 98% and 50%, respectively. Accordingly, 50% of the samples were considered unusable. They also noted that the presence of *E*. *coli* could increase the possibility of the existence of other pathogens in raw milk cheese. Since the presence of *E*. *coli* makes the cheese inedible, due to the high contamination of local Iranian cheeses more than foreign ones, it is necessary to observe some principles in the preparation and storage of Lighvan cheese to inhibit the high microbial contamination, which includes (i) heating of raw milk above 50℃, (ii) observing hygienic standards during production, (iii) using appropriate strains of Lactic starters, (iv) using salt, and (v) prolonging the ripening time (Saidi et al., 2020).

Similar to the raw milk cheeses, the pasteurized samples showed a reduction in the microbial population during the ripening period, in which the total bacteria count decreased from 6.6 log CFU/gr to 4.81 log CFU/gr at the last day of storage period. However, due to the thermal treatment of milk, the count of total bacteria was significantly lower than that of raw milk cheeses. Also, the obtained results indicated a significant effect of thermal treatment of milk on the count of all microbial groups (*p* < .05). Pasteurization lowered the number of *Enterococci, Micrococcus*, and lactic acid bacteria. It also eliminated coliforms, *Lactobacilli*, yeasts, *E*. *coli, and Listeria*. However, the *Micrococcus* and lactic acid bacteria populations remained stable (*p* ˃ 0.05) until the last day of ripening time.

### 3.2 Physicochemical characteristics

The physiochemical properties of two types of Lighvan cheeses (raw and pasteurized) over the ripening period are exhibited in Table 2. PH values showed no significant (*p* > .05) changes over the time in both samples. This is in opposition to the study of Aminifar et al. (2014) showed a reduction in pH value of Lighvan cheese during the ripening period. The reduction in pH is attributed to fermentation of lactose and changing it to lactic acid, as well as the liberation of free fatty and amino acid by proteolysis and lipolysis (Lavasani et al., 2012). Whereas, its increase may associated with the catabolism of lactic acid by microorganisms such as yeasts and its migration to the brine thorough ripening, as well as the production of ammonia following proteolysis. According to the obtained results, it seems that showing constant pH over time may attribute to the fact that the rate of lactose fermentation, proteolysis, and lipolysis reactions are the same, which neutralizes their effect on the pH of cheese samples. Also, it may attribute to the association of lactic acid bacteria to fat globules due to agglutinin activity and rising with cream over resting of milk, which may in turn, inhibit acidification (Psoni et al., 2006). The comparison of the pH values between two types of cheeses exhibited a lower pH value for raw milk cheeses compared with the pasteurized one (Table 2). It may attribute to the elimination of native enzymes, and microflora present in raw milk, responsible for pH decrease in cheese during ripening by heat treatment during pasteurization. Similar results have reported by Saidi et al. (2020); Psoni et al. (2003); and Awad (2006) for Lighvan, Greek, and Ras cheeses, respectively. In the study, Aminifar et al. (2014) showed that carboxylic acids are the most important compounds produced over the cheese ripening process. Therefore, reduce the pH of ripened cheese is mostly attributed to the production of carboxylic acid compounds.

**TABLE 2:** Chemical properties of Lighvan cheese during ripening

Ripening time	cheese	Day 2	Day 14	Day 35	Day 60
Dry matter (g/kg)	Raw	39.38 ± 0.65^a,b^, ^A,B,C^	40.37 ± 0.41^a,b^, ^A,B,C^	41.26 ± 0.53^a,b^, ^A,B,C^	41.59 ± 0.51^a,b^, ^A,B,C^
	Pasteurized	39.44 ± 0.21^a,b^, ^A,B,C^	39.89 ± 0.73^a,b^, ^A,B,C^	41.06 ± 0.23^a,b^, ^A,B,C^	40.87 ± 0.7^a,b^, ^A,B,C^
Fat (g/kg dry matter)	Raw	19.21 ± 0.51^a,b^, ^A,B,C^	19.31 ± 0.32^a,b^, ^A,B,C^	20.73 ± 0.33^a,b^, ^A,B,C^	21.51 ± 0.61^a,b^, ^A,B,C^
	Pasteurized	18.34 ± 0.13^a,b^, ^A,B,C^	19.00 ± 0.45^a,b^, ^A,B,C^	19.99 ± 0.83^a,b^, ^A,B,C^	20.53 ± 0.71^a,b^, ^A,B,C^
pH	Raw	5.55 ± 0.02^a,b^, ^A,B,C^	5.52 ± 0.02^a,b^, ^A,B,C^	5.40 ± 0.01^a,b^, ^A,B,C^	5.36 ± 0.02^a,b^, ^A,B,C^
	Pasteurized	6.11 ± 0.02^a,b^, ^A,B,C^	6.22 ± 0.02^a,b^, ^A,B,C^	6.14 ± 0.02^a,b^, ^A,B,C^	6.36 ± 0.02^a,b^, ^A,B,C^
NaCl (%)	Raw	7.16 ± 0.18^A,B,C^	7.43 ± 0.15^a,b^, ^A,B,C^	7.81 ± 0.15^a,b^, ^A,B,C^	8.09 ± 0.18^a,b^, ^A,B,C^
	Pasteurized	6.87 ± 0.15^a,b^, ^A,B,C^	7.99 ± 0.15^a,b^, ^A,B,C^	7.25 ± 0.14^a,b^, ^A,B,C^	8.15 ± 0.12^a,b^, ^A,B,C^

^a,b^ Means in the same raw, the same superscript does not differ significantly at *p* < .05.

^A,B,C^ Different superscripts in the same column indicate significant differences (*p* < .05)

[Correction added on August 13, 2021 after first online publication: the table footnote (* p < .05; ** p < .01; *** p < .001; ns p ˃ .05) has been removed]

The salt and total solid content of raw and pasteurized types of cheese over ripening period are shown in Table 2. The salt content increased significantly (*p* < .05) in both kinds of cheese over time, attribute to the NaCl diffusion from the brine to the cheese body. The raw cheese indicated higher NaCl content than the pasteurized one, which may attribute to the smoother texture of raw cheese compared with the other one. Probably due to the formation of β‐Lactoglobulin—κ‐Casein complex, the texture of pasteurized cheese is firmer, which shows greater resistance to the penetration of salt into its texture compared with the raw sample. Similar results have shown by Morandi et al. (2020). The results of total solid content indicated a significant increase (*p* < .05) in both kinds of cheese over the storage time, attributed to the diffusion of NaCl molecules from the brine to the texture of cheese (Farahani et al., 2014). Comparison between two types of cheeses showed a higher dry matter for raw cheese than the other one, which attributes to the formation of β‐lg‐casein complex, which modifies the surface properties as well as the reaction between casein micelles (Singh & Waungana, 2001). Similarly, Psoni et al. (2006) have shown an increase in solid content of Batzos cheese throughout ripening.

The fat content increased slightly over ripening time in both kinds of cheeses. This phenomenon attributes to the increase in the dry matter after placing the cheese into the brine during ripening, which is parallel to moisture content reduction that may lead to an increase in fat content. The comparison of the two samples indicated the higher fat content for raw milk cheese than the pasteurized one. This is related to the denaturation of whey proteins and their subsequent binding to k‐casein over heat treatment in the pasteurization process, may lead to more whey protein retention, and destruction of the fat globule membranes may also lead to a reduction in the fat content of pasteurized milk cheeses (Morales‐Celaya et al., 2012; Xiong et al., 2020).

### 3.3 Lipolysis

**FIGURE 2:**
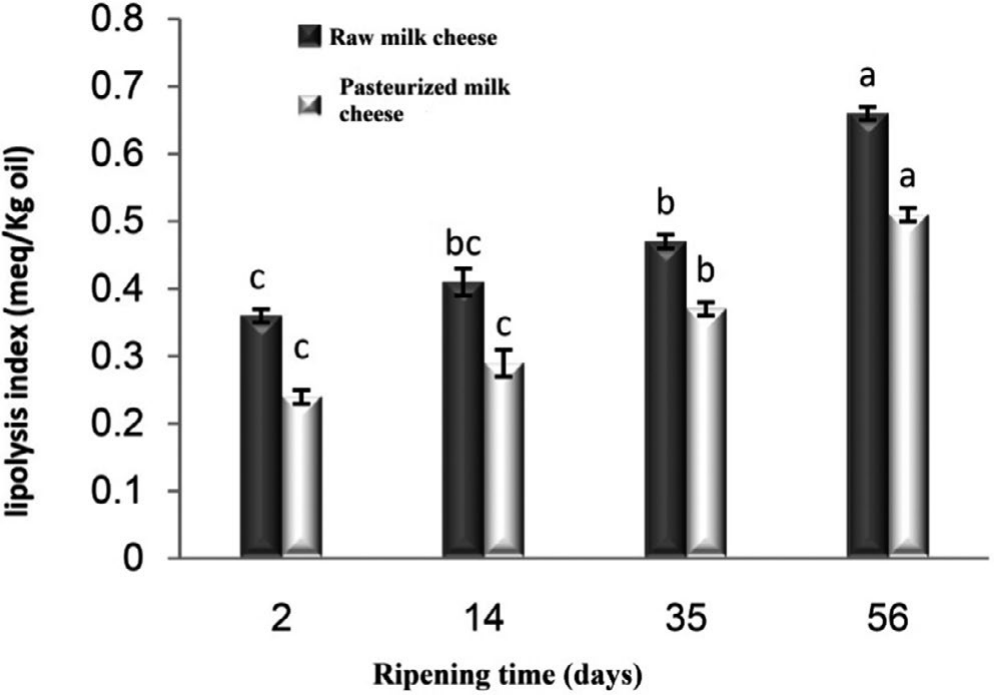
Lipolysis index in Lighvan cheese made of raw and pasteurized milk

Cheese ripening is a very important process in which lipolysis, proteolysis, and glycolysis reactions play an essential role in the development of the organoleptic properties of the product. Lipolysis is a major biochemical reaction in cheese production that plays a key role in the development of the aroma and flavor of cheese due to the production of volatile free fatty acids through the activity of indigenous milk lipase (Mc‐Sweeney & Sousa, 2000). The obtained results from the evaluation of lipolysis throughout ripening time are presented in Figure 2. The lipolysis index showed an increasing trend in both types of cheeses over time. The raw milk cheese illustrated a higher lipolysis index (*p* < .05) than the pasteurized one. The native milk lipoprotein lipase (LPL) and lipases of indigenous non‐starter lactic acid bacteria may have contributed to a higher level of free fatty acids in raw milk cheese than pasteurized one. However, contrary to these results, a decrease in lipolysis due to decreased lipase activity after 30 days of storage in cheese was reported due to changes in environmental conditions such as pH and moisture content (Buffa et al., 2001).

### 3.4 Proteolysis

**FIGURE 3:**
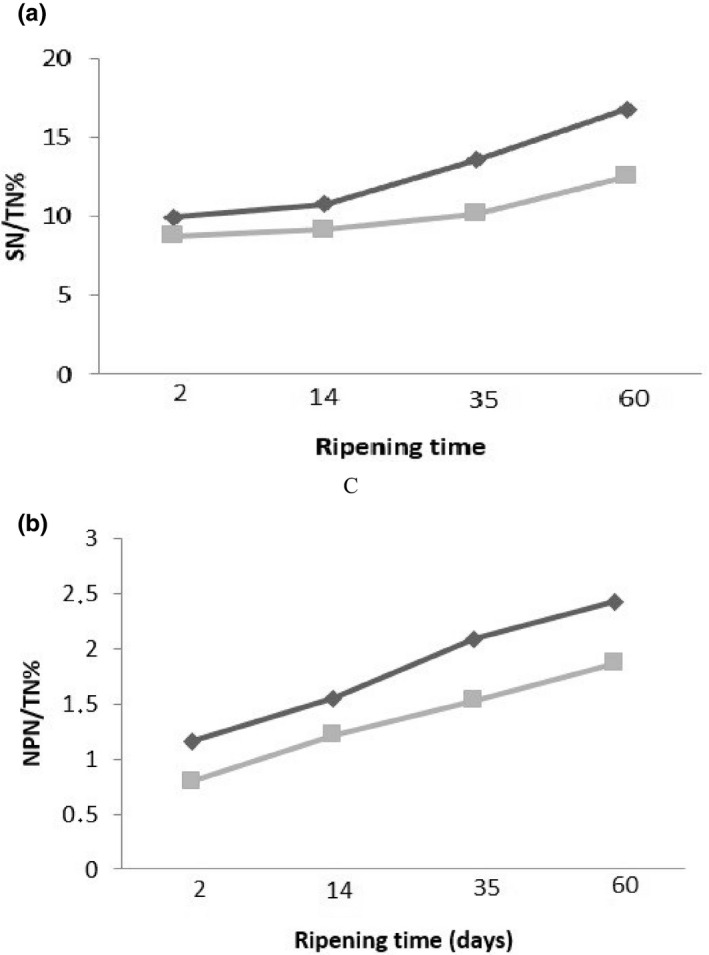
PH 4.6 soluble nitrogen (SN) as the percentage of total nitrogen (TN) (a), and non‐protein nitrogen (NPN) as the percentage of total nitrogen (TN) (b) in Lighvan cheese made of raw (■) and pasteurized milk (♦)

In addition to lipolysis, another important biochemical process that occurs over ripening of most types of cheese is proteolysis. Due to the production of amino acids and low molecular weight peptides which are flavored and/or by providing free amino acids which are substrates for some catabolic reactions may lead to generate some flavored compounds, proteolysis has a key role in the development of the sensory properties of cheese. The origin of proteolytic enzymes included the coagulant, the milk (plasmin), starter LAB, NSLAB, secondary starters, Gram‐positive bacterial microflora, and exogenous enzymes added to the milk or curd to accelerate ripening (Mc‐Sweeney, 2004). Figure 3 (A–B) shows the results of the pH 4.6 soluble nitrogen SN/TN and non‐protein nitrogen NPN/TN, respectively. The pH 4.6 soluble nitrogen contains numerous small and medium‐sized peptides and free amino acids. Their degraded products are produced mainly by the coagulants, and to a lesser extent, plasmin. The produced cheese from raw milk indicated a further increase in pH 4.6 soluble nitrogen than another sample, where this factor increased from 9.95 to 16.8 after 60 days in this type of cheese. However, two cheese samples from raw and pasteurized milk did not show any difference. The proteinases and peptidases from lactic acid bacteria are responsible for the production of non‐protein nitrogen as shown in Figure 3‐B. The level of NPN/TN increased during ripening and indicated a lower level for pasteurized cheese.

**FIGURE 4:**
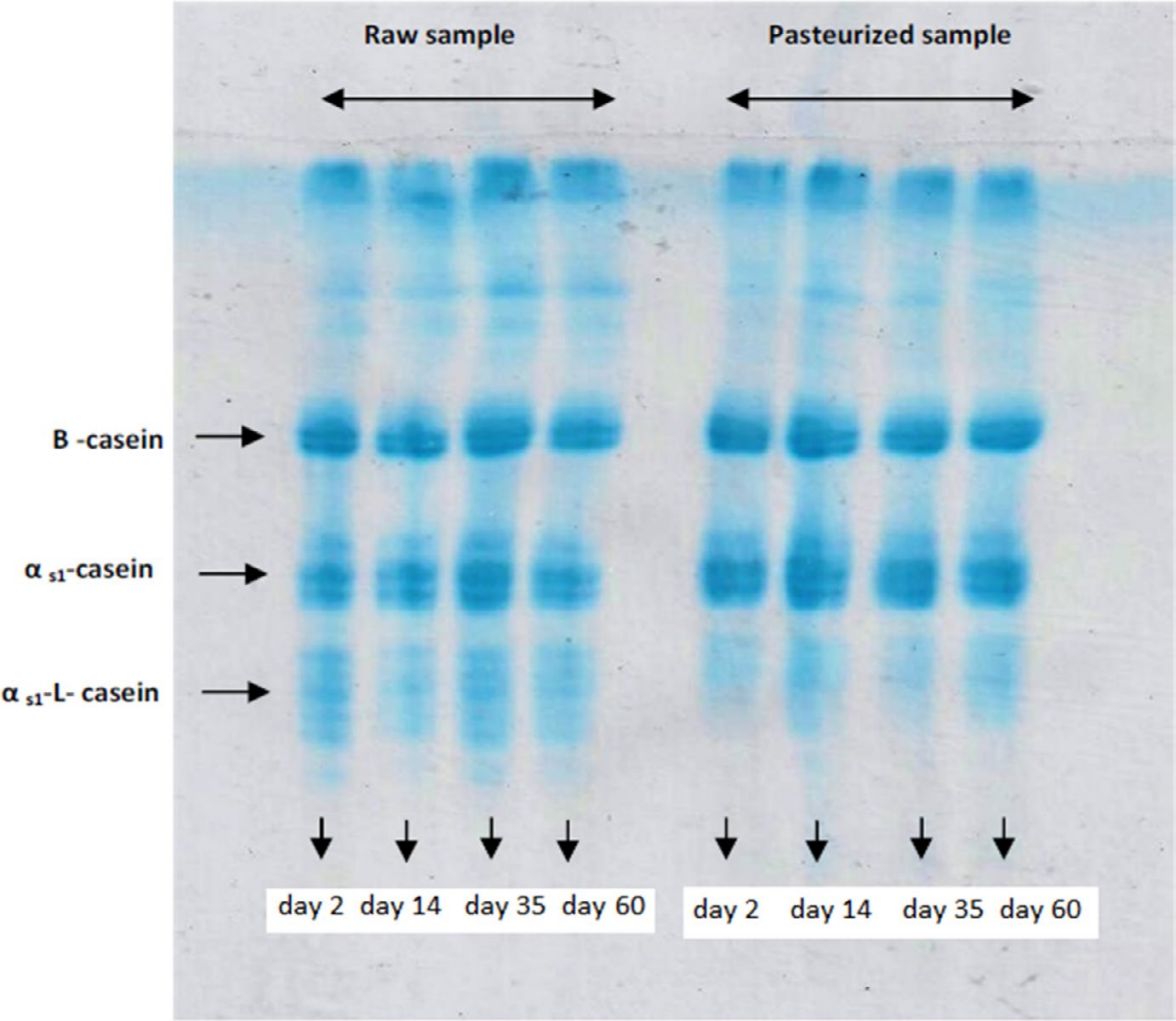
Urea‐PAGE profiles of Lighvan cheese made from raw and pasteurized milk, after 2, 14, 35, and 60 days of storage

Electrophoretograms of both types of cheeses over ripening are exhibited in Figure 4. The results indicated higher proteolysis in raw milk cheese compared with the pasteurized one. The intensity of the band attributes to ß‐casein and αs_1_‐casein reduced slowly over storage time with a simultaneous increase in the bands correspond to γ‐caseins and αs_1_‐l‐casein, since these are the product of the decomposition of ß‐casein and αs_1_‐casein, respectively. The amount of αs_1_‐casein breakdown products in raw milk cheese is higher than the heated one. Due to having more non‐starter lactic acid bacteria and plasmin enzyme as well as the presence of coliforms and yeasts, the raw milk cheese shows more degradation of casein micelles compared with pasteurized milk cheese. Differences in casein break down between the two types of cheeses may be related to the presence of heat‐denatured whey proteins in pasteurized milk that influences the accessibility of proteinases and the action of non‐starter peptidases present in the raw milk cheeses.

### 3.5 Sensory Properties

**FIGURE 5:**
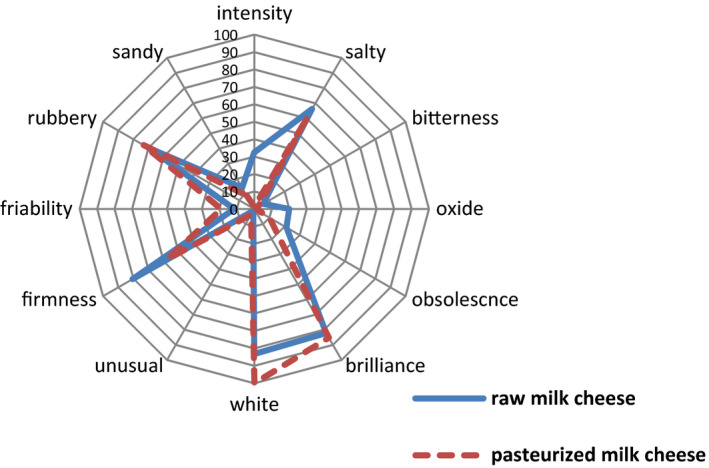
Sensory characteristic of cheese measured at the end of ripening

The sensory properties of two types of cheeses on the last day of ripening (day 60) and the analysis of variance are presented in Figure 5 and Table 3. According to Figure 5, the saltiness, bitterness, obsolescence taste, unusual flavor, friability, rubbery, and sandy were not affected by the pasteurization of milk, but the intensity was lower in pasteurized cheese (*p* < .01). Oxidization and firmness were drastically influenced by thermal treatment (*p* < .05). We determined also a significant difference (*p* < .001) in white color between the two types of cheeses. The raw milk cheese generally has a stronger flavor than the pasteurized one and reaches an optimum flavor faster. However, in a study by Buffa et al. (2001), cheese obtained from pasteurized milk scored higher due to the excessive production of free amino acids during the lipolysis process over ripening period of raw milk cheeses.

**TABLE 3:** The Sensory characteristic of cheese measured at the end of ripening

Freedom Resources	1	
Freedom Degree		1
	Intensity	4,867.2^**^
	Salty	125.00^ns^
	Bitterness	61.25^ns^
mean square	Oxide	1,757.81^*^
	Obsolescence	627.20^ns^
	Brilliance	45.00^ns^
	Firmness	1,411.20^*^
	Unusual taste	31.20^ns^
	White	2,645.00^***^
	Friability	281.25^ns^
	Rubbery	6.25^ns^
	Sandy	125.00^ns^

* *p* < .05; ** *p* < .01; *** *p* < .001; ns *p* ˃ .05.

## 4 CONCLUSIONS

The obtained results showed that the heat treatment of cheese milk significantly reduces bacteria counts and eliminates pathogen bacteria together with many other bacteria. According to the results of sensory evaluation, the raw milk cheese has a better flavor and, also overall acceptance than pasteurized milk cheese. Because *E*. *coli* was detected in some samples after 60 days, it is suggested that 60 days is not sufficient to vanish pathogen microflora in raw milk cheese. Therefore, pasteurization of cheese milk is mandatory to ensure its safety. It is also possible to add lactic bacteria to develop and enhance a pleasant Lighvan cheese flavor in the pasteurized cheese.
